# 

**Published:** 1928

**Authors:** 


### CONTENTS.

| | Page |
|---|---|
| The Long Fox Memorial Lecture: The Relationship of Erythema Nodosum to Tuberculosis. By J. ODERY SYMES, M.D. | 233 |
| The Early Diagnosis and Treatment of Insanity. By J. V. BLACHFORD, M.D. | 253 |
| Peri-Arterial Sympathectomy. By E. W. HEY GROVES, M.S., F.R.C.S. | 273 |
| The Symptoms and Signs of Duodenal Ulcer. By T. CARWARDINE, M.S., F.R.C.S. | 279 |
| The Medical Treatment of Duodenal Ulcer. By R. C. CLARKE, M.B., M.R.C.P. | 289 |
| Treatment of Infantile Paralysis of the Lower Limb. By M. FORRESTER BROWN, M.S., M.D. Lond. | 295 |
| Reviews of Books | 309 |
| Editorial Notes | 319 |
| Obituary: FRANK J. WETHERED, M.D. (Lond. and Bristol), F.R.C.P. | 321 |
| Meetings of Societies | 322 |
| The Medical Library of the University of Bristol | 324 |
| Local Medical Notes | 326 |

